# Bisdemethoxycurcumin and Curcumin Alleviate Inflammatory Bowel Disease by Maintaining Intestinal Epithelial Integrity and Regulating Gut Microbiota in Mice

**DOI:** 10.1021/acs.jafc.4c11101

**Published:** 2025-01-28

**Authors:** Kai-Yu Hsu, Anju Majeed, Chi-Tang Ho, Min-Hsiung Pan

**Affiliations:** † Institute of Food Sciences and Technology, National Taiwan University, Taipei 10617, Taiwan; ‡ Sami-Sabinsa Group Limited, Bengaluru, Karnataka 560058, India; § Department of Food Science, Rutgers University, New Brunswick New Jersey 08901 United States; ∥ Department of Medical Research, China Medical University Hospital, China Medical University, Taichung 40402, Taiwan

**Keywords:** curcumin, bisdemethoxycurcumin, inflammatory bowel disease, tight junction, microbiota

## Abstract

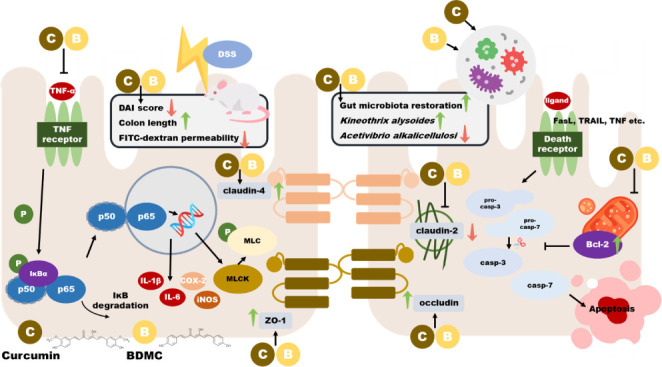

Curcuminoids, found in turmeric (*Curcuma longa* L.), include curcumin (CUR), demethoxycurcumin (DMC), and bisdemethoxycurcumin (BDMC). Although CUR and DMC are well-studied, the anti-inflammatory effects of BDMC remain less explored. Recent studies highlight BDMC’s stronger NF-κB inhibition compared to CUR and DMC in cell models, along with its ability to target pathways associated with inflammatory bowel disease (IBD) in DSS-induced colitis mice, reflected by lower disease activity scores and reduced inflammation. This study assessed CUR and BDMC in a DSS-induced colitis mouse model. Dietary administration of CUR or BDMC strengthened tight junction (TJ) proteins, reduced inflammatory cytokine secretion, and attenuated intestinal inflammatory protein expression, thereby alleviating DSS-induced IBD in mice. Furthermore, gut microbiota and short-chain fatty acid analyses revealed that CUR and BDMC effectively regulated gut microbial imbalance and promoted the relative abundance of butyrate-producing bacteria. Furthermore, CUR showed low absorption and was primarily excreted in feces, while BDMC had higher absorption levels. In conclusion, while both BDMC and CUR have potential as adjunct therapies for IBD, BDMC at a concentration of 0.1% showed strong anti-inflammatory effects and enhanced TJ proteins, suggesting that BDMC, even at lower concentrations than CUR, holds promising therapeutic potential and prospects.

## 1 Introduction

Inflammatory bowel disease (IBD) refers to a group of chronic inflammatory diseases affecting the digestive tract. Depending on the location and severity of the inflammation, it can be categorized as ulcerative colitis (UC), Crohn’s disease, and indeterminate colitis.^1^ IBD can cause symptoms such as abdominal pain, diarrhea, bloody stools, anemia, and weight loss due to malabsorption.^2^ It may also lead to damage to the surface tissue, and in severe cases, there is a risk of intestinal rupture or perforation. The newly formed mucosa after inflammation may develop into raised areas within the intestinal wall known as pseudopolyps or lead to complications such as intestinal fibrosis and fistulas. Additionally, prolonged intestinal inflammation can cause abnormal mucosal growth, increasing the risk of developing colorectal cancer.^3^ When the intestinal barrier is compromised due to genetic or external environmental factors, its permeability increases, making it easier for gut bacteria to translocate. This allows microbial products, such as endotoxins and bacterial metabolites, to migrate from the intestinal lumen to the intestinal wall. These microbial products are recognized by immune cells, such as macrophages and lymphocytes in the lamina propria, triggering further immune responses.^4^

However, when a large number of immune cells gather and an excess of cytokines is produced, the balance of the internal intestinal environment can be disrupted, potentially leading to acute enteritis.^5^ In the early stages of enteritis, the released cytokines help restore the health of the intestinal barrier, but if the body’s anti-inflammatory response is insufficient to alleviate the condition, the persistent cytokines will infiltrate and damage the intestinal barrier, eventually leading to chronic IBD.^6^ The inflammatory response is originally a defense mechanism of the body designed to remove harmful substances and repair tissues. This process involves various immune cells, such as monocytes and macrophages, which produce proinflammatory cytokines like tumor necrosis factor alpha (TNF-α), interleukin-1 beta (IL-1β), and interleukin-6 (IL-6). These cytokines then stimulate corresponding receptors, activating related inflammatory pathways, such as mitogen-activated protein kinase (MAPK), nuclear factor kappa-light-chain-enhancer of activated B cells (NF-κB), and Janus kinase-signal transducer and activator of transcription, thereby promoting or inhibiting inflammation to protect and repair damaged tissue.^7^ However, when this mechanism is disrupted, regulatory T cells may fail to produce sufficient interleukin-10 (IL-10) or may not properly activate transforming growth factor-beta, leading to an imbalance in the immune response.^8^

Turmeric (*Curcuma longaL.*) is a medicinal plant widely used in traditional Chinese and Indian medicine and is cultivated around the world, including in regions such as Southeast Asia, China, and Latin America.^9^ The turmeric rhizome has been used for centuries to treat liver diseases and other inflammation-related symptoms,^10^ with functional activities mainly attributed to its rich curcuminoid content.^9,11^ Curcuminoids are linear diarylheptanoids that include curcumin (CUR). Two related compounds, demethoxycurcumin (DMC) and bisdemethoxycurcumin (BDMC), of curcuminoids mainly differ in the presence or absence of methoxy groups at the 2 and 2’ carbon positions. Previous studies have indicated that curcuminoids possess strong *in vitro* antioxidant capabilities. However, BDMC is an exception. Lacking methoxy functionality, its antioxidant capacity is significantly reduced. The antioxidant capacities of the curcuminoids were in the following order: curcumin > demethoxycurcumin > bisdemethoxycurcumin.^12^ Therefore, the compound has been largely overlooked in the past. Curcuminoids have demonstrated their ability to reduce NF-κB activity in LPS-induced RAW264.7 cells transfected with luciferase, with BDMC showing the most significant effect. Additionally, curcumin and DMC inhibit NF-κB through their oxidation into an electrophilic species. In contrast, due to the lack of an electron-donating adjacent methoxy group, BDMC is insensitive to spontaneous oxidative conversion.^13^ This also explains why curcumin and DMC share similar inhibitory pathways in most of the literature, whereas BDMC does not.

Studies have indicated that consuming turmeric powder or curcumin in the diet can exert protective effects against IBD by reducing the level of activation of inflammatory pathways, improving gut microbiota dysbiosis, promoting microbial metabolism, and repairing intestinal barrier damage. A recent study has identified potential targets and molecular mechanisms of BDMC in IBD, indicating that BDMC may reduce the release of proinflammatory cytokines by potentially targeting nonreceptor tyrosine kinase (SRC), epidermal growth factor receptor (EGFR), AKT threonine-protein kinase (AKT1), and phosphoinositide-3-kinase regulatory subunit 1 (PIK3R1), with notable effects on the PI3K/AKT and mitogen-activated protein kinase (MAPK) pathways. However, the findings have yet to be validated through animal experiments.^14^ In this study, we further explored the efficacy of curcumin and BDMC using a dextran sulfate sodium (DSS)-induced colitis mouse model. We also compared curcumin and BDMC in a 5:1 concentration ratio, mimicking their proportions in turmeric, to determine whether the lower concentration of BDMC in turmeric has similar effects.

## 2 Materials and Methods

### 2.1 Chemicals, Reagents, and Antibodies

Curcumin, bisdemethoxycurcumin (BDMC), tetrahydrocurcumin (THC), hexahydrocurcumin (HHC), and feruloylacetone (FER) (with a purity of >99%, as determined by high-performance liquid chromatography) were provided by Sabinsa Corporation (East Windsor, NJ, USA). Antibodies against NF-κB p65 and p-MLC were obtained from Cell Signaling Technology (Beverly, MA, USA). Antibodies targeting Bcl-2, Bax, claudin-4, MLCK, and occludin were sourced from Proteintech (Rosemont, IL, USA). Antibodies against NF-κB p-p65 and claudin-2 were purchased from Santa Cruz Biotechnology (Dallas, TX, USA). Antibodies against COX-2 were purchased from BD Bioscience (East Rutherford, NJ, USA). Antibodies against iNOS were purchased from Abcam PLC (Cambridge, UK). Secondary antibodies were supplied by GeneTex (Irvine, CA, USA) and Abcam PLC (Cambridge, UK). Fluorescein isothiocyanate (FITC)-dextran was obtained from Sigma Chemical Corporation (St. Louis, MO, USA).

### 2.2 Animal Experiment Design

A mouse model of DSS-induced colitis was established following methods previously described.^15^ Forty male Institute of Cancer Research (ICR) mice were obtained from the BioLASCO Experimental Animal Center (Taipei, Taiwan). The study protocols were reviewed and approved by the Institutional Animal Care and Use Committee of National Taiwan University (NTU-111-EL-00081). Mice were housed at 25 ± 1 °C with 50% relative humidity and a 12-h light-dark cycle. After a 1-week acclimation period, the mice were randomly divided into five groups (*n* = 8 per group): (1) control (CON, normal diet), (2) DSS-induced colitis (IND, normal diet + 2% DSS solution), and (3–5) curcuminoid groups with DSS-induced colitis. Referring to the minimum effective dose from a previous study,^16^ two groups were provided with 2% DSS solution + normal diet supplemented with 0.5% CUR and 0.5% BDMC, respectively. Additionally, the natural ratio of curcumin to BDMC in turmeric was simulated,^17^ and a low-dose group with a normal diet + 0.1% BDMC was evaluated. Two cycles of 2% DSS solution (MP Biomedicals, LLC, Illkirch, France) were administered, with a 7-day treatment followed by a 14-day interval of deionized water. The mice had access to food and water ad libitum. Curcuminoid diets were introduced 1 week prior to DSS treatment as a pretreatment. Body weight and disease activity index (DAI) were measured regularly during the study period. DAI was evaluated based on clinical symptoms such as weight loss, stool consistency, and the presence of hematochezia. At the end of the study period, all mice were euthanized using CO_2_ and dissections were performed. Blood samples were collected via cardiac puncture and centrifuged to separate the plasma. The spleen was harvested and weighed, and the entire colon was dissected from the cecum to the anus, weighed, and photographed, with lengths recorded. All samples were stored at −80 °C for further analysis.

### 2.3 Intestinal Permeability Assessment *In Vivo*

Intestinal permeability was evaluated by quantifying the amount of FITC-dextran in the bloodstream after oral administration, following established protocols.^15^ Briefly, each mouse received a gavage of FITC-dextran (molecular weight 4 kDa, Sigma-Aldrich) at a dose of 400 mg/kg. Two hours later, blood samples were collected and centrifuged at 10,000 g for 10 min at 4 °C. The serum was then isolated and transferred to a 96-well microplate. The FITC-dextran concentration in the serum was determined using photofluorometry, with excitation and emission wavelengths set at 485 and 528 nm, respectively.

### 2.4 Western Blot Analysis

Tissue samples (scraped colon mucosa) were homogenized in ice-cold lysis buffer, vortexed on ice for 1 h, and centrifuged at 14,000 g for 30 min at 4 °C. Protein concentrations were determined using a Bio-Rad protein assay. Total protein (40 μg) was loaded onto a 10% sodium dodecyl sulfate-polyacrylamide gel for electrophoresis and then transferred onto PVDF membranes (Merck Millipore Ltd., Tullagreen, County Cork, Ireland). Membranes were blocked with a 5% BSA solution and incubated overnight at 4 °C with primary antibodies against the target proteins (Bax, Bcl-2, claudin-2, claudin-4, COX-2, i-NOS, MLCK, NF-κB p65, p-p65, occludin, and p-MLC). The next day, membranes were incubated for 1 h at room temperature with horseradish peroxidase (HRP)-conjugated secondary antibodies. Protein bands were visualized using enhanced chemiluminescence (ECL) and documented with the Multi Gel system. Protein band densities were quantified using ImageJ software, with β-actin serving as the loading control.

### 2.5 Measurement of Proinflammatory Cytokines

Proinflammatory cytokine levels in colon homogenates were measured using a commercial ELISA kit (Invitrogen, Waltham, MA, USA) following the manufacturer’s instructions. Specifically, TNF-α (catalog no. 88-7324), IL-1β (catalog no. 88-7013), and IL-6 (catalog no. 88-7064) were analyzed using an ELISA reader (BioTek Instruments, Winooski, VT, USA). Cytokine levels were normalized to the colon tissue protein concentrations.

### 2.6 Measurement of Epithelial Cell Apoptosis in the Colonic Tissue

Cleaved caspase-3 and caspase-7 levels in colon homogenates were measured using a commercial protein array kit (catalog no. AAM-APO-1-8, RayBiotech, Peachtree Corners, GA, USA) following the manufacturer’s instructions. Protein levels were normalized to the colon tissue protein concentrations. Protein bands were visualized using ECL and documented with the Multi Gel system. Protein spot densities were quantified by using ImageJ software.

### 2.7 Gut Microbiota Analysis

Collection of fecal samples and gut microbiota analysis were performed following previously described methods.^18^ Genomic DNA from gut bacteria was isolated and purified using an InnuPREP Stool DNA kit, with slight modifications. DNA samples were sent to Biotools Co. Ltd. (Taipei, Taiwan) for fecal microbial composition analysis via 16S amplicon sequencing. PCR was used to amplify the 16S rRNA gene, including 10 conserved regions (V3–V4) and 9 hypervariable regions (V1–V9). Sequencing on an Illumina HiSeq2500 platform (250 bp) generated reads that formed effective tags clustered into amplicon sequence variants (ASVs) at a 97% identity threshold, representing bacterial species or genus. Weighted and unweighted UniFrac analyses were conducted to quantify indices such as PCA (principal component analysis) or PCoA (principal coordinate analysis), while LEfSe (linear discriminant analysis effect size) identified the most significant differences among samples.

### 2.8 HPLC–MS/MS and GC–MS Analysis of SCFAs and Curcuminoids in Experimental Samples

A total of six short-chain fatty acid (SCFA) standards (acetic acid, propionic acid, isobutyric acid, butyric acid, isovaleric acid, and valeric acid), along with 4-methylvaleric acid (internal standard), were dissolved in ethyl acetate to prepare stock solutions. For GC–MS analysis, a DB-WAXetr capillary column was used with helium as the carrier gas at a flow rate of 1 mL/min in splitless mode. The following conditions were applied: injector temperature 250 °C, MS interface temperature 280 °C, and an oven temperature program starting at 90 °C, increasing to 150 °C at a rate of 15 °C/min, then increasing to 170 °C at 5 °C/min, finally increasing to 250 °C at 20 °C/min, and holding for 2 min. The selected ion monitoring (SIM) mode was employed for detection based on the elution order and appropriate *m*/*z* ratios, as outlined in a previous study.^19^ A total of five curcuminoid standards, including curcumin, DHC, BDMC, THC, and HHC, along with methyl red (internal standard), were individually dissolved in acetonitrile to create stock solutions, each at a concentration of 1000 ng/mL. HPLC–MS/MS analysis was performed by using a ZORBAX Eclipse XDB-C18 column. The mobile phase consisted of A (0.1% formic acid) and B (acetonitrile), with the gradient starting at 40% mobile phase B for 2 min. The ratio was then increased to 90% B over 18.75 min and maintained for 2 min. The column temperature was maintained at 25 °C with a flow rate of 0.7 mL/min. Detection was performed using a triple quadrupole tandem mass spectrometer (QqQ) equipped with electrospray ionization (ESI) in the selected reaction monitoring (SRM) mode.

### 2.9 Statistical Analysis

Data are presented as mean ± standard error (SEM). The Student’s *t*test and one-way ANOVA with Duncan’s multiple comparison tests were performed to detect significant differences between groups at a significance level of *p* < 0.05. Sample sizes ranged from 3 to 4 for Western blot analysis, with up to 8 samples included in other analyses. Data points were excluded if they deviated by two or more standard deviations from the mean. This discrepancy in sample sizes across analyses was due to the need to reserve sufficient tissue and serum for all studies, with a minimum of three samples per group.

## 3 Results and Discussion

IBD may stem from intestinal barrier dysfunction triggered by genetic and environmental factors. Westernized diets high in fats and sugars, low fiber, reduced vitamin D, stress, lack of exercise, poor sleep, and antibiotic misuse may all increase IBD risk.^51^ Given the current treatment limitations, exploring new dietary strategies is essential.

Curcumin is a promising candidate for managing IBD due to its anti-inflammatory and antioxidant properties. Studies have shown that curcumin reduces neutrophil infiltration at inflammatory sites by disrupting chemokine gradients and directly affecting neutrophil functions. This protective effect is crucial during intestinal inflammation. Curcumin also controls inflammation by downregulating genes related to oxidative stress and fibrogenesis, while inhibiting proinflammatory cytokines and key signaling pathways like NF-κB and MAPK.^14^ BDMC is another curcuminoid compound derived from turmeric. However, because BDMC lacks methoxy groups, its phenolic ring structure cannot engage in neighboring group participation with hydroxyl groups, resulting in the loss of its antioxidant activity.^52^ This has led to a relative lack of research interest in BDMC. However, recently, studies have shown that BDMC exhibits superior anti-inflammatory effects.^53^ Indeed, BDMC may reduce LPS-induced proinflammatory cytokine levels in RAW264.7 cells by inhibiting the PI3K/Akt and MAPK pathways, and several promising targets have also been identified among the intersecting genes.^14^ However, few studies have investigated the mechanisms by which BDMC alleviates colitis, and no research has been conducted on BDMC’s effect on reducing DSS-induced colitis in mice. Therefore, in this study, a DSS-induced colitis mouse model was used to evaluate the effects of BDMC and curcumin on reducing inflammation and enhancing intestinal barrier function. Additionally, because BDMC is present at approximately one-fifth the concentration of curcumin in turmeric rhizomes,^17^ the efficacy of 0.1% BDMC was also assessed alongside the comparison between 0.5% curcumin and 0.5% BDMC.

### 3.1 BDMC and Curcumin Ameliorated Chronic Colitis Induced by DSS

**Figure 1:**
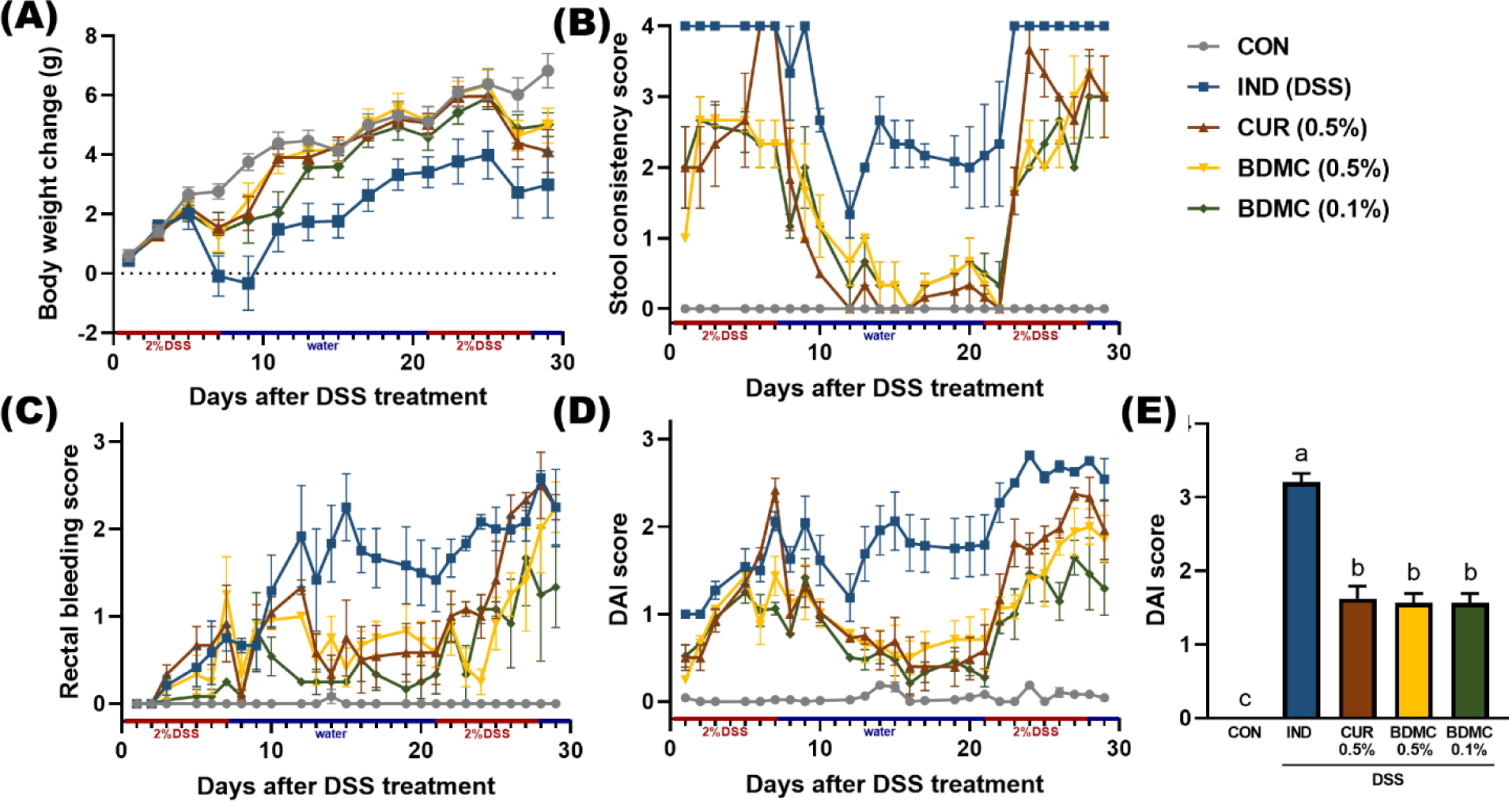
Effects of BDMC and curcumin on disease activity index (DAI) in DSS-induced ICR mice. The DAI score is the average of three individual parameters, combining (A) weight loss, (B) stool consistency, and (C) rectal bleeding into one score. DAI scores were measured every 2 days after DSS administration until the end of experiment. (D) DAI score and (E) average DAI score. Data are presented as mean ± SEM, *n* = 8 per group, and *p* values were determined by one-way ANOVA with subsequent Duncan’s multiple comparison test. The values with different letters are significantly different (*p* < 0.05) between each group.

**Figure 2:**
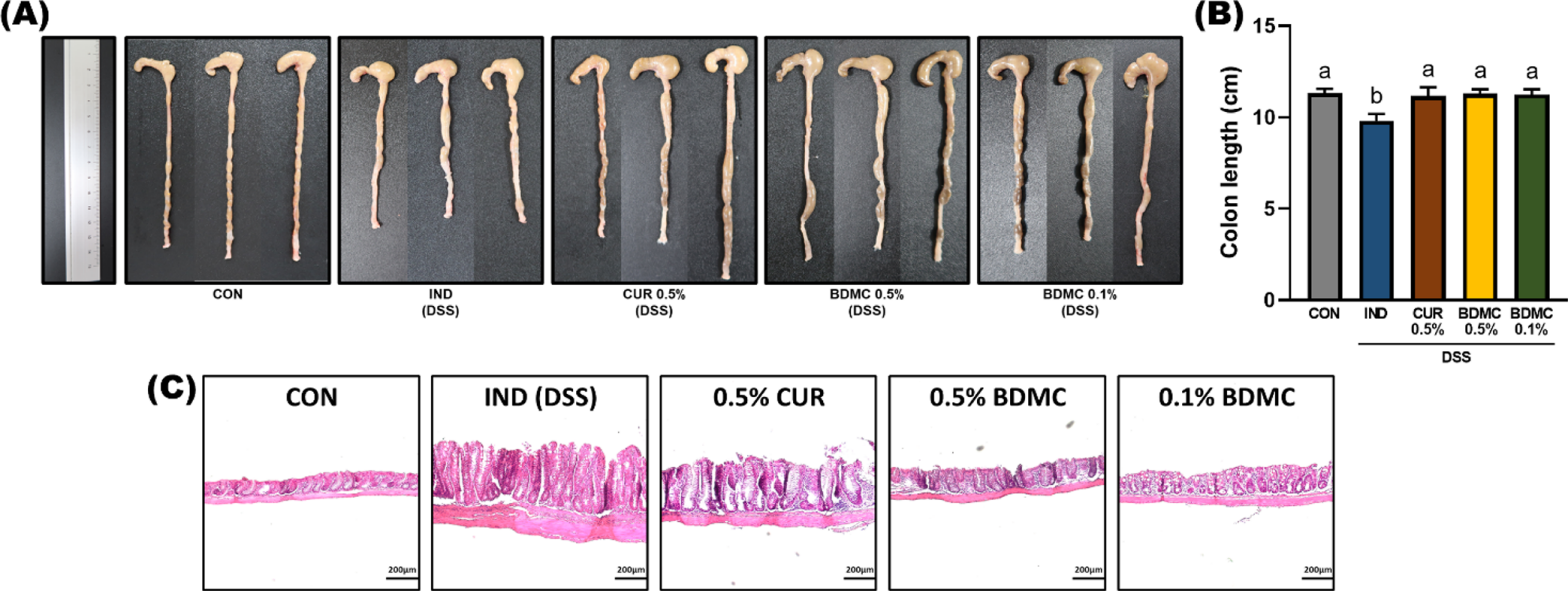
Effects of BDMC and curcumin on colon length and morphology in DSS-induced ICR mice. (A) Representative macroscopic appearances of colon tissue. (B) Measurement of colon length. Data are presented as mean ± SEM, *n* = 8 per group, and *p* values were determined by one-way ANOVA with subsequent Duncan’s multiple comparison test. The values with different letters are significantly different (*p* < 0.05) between each group. (C) Representative photomicrographs from H&E-stained 4 μm colonic tissue sections at 100 × magnification (scale bar: 200 μm).

The DAI score, which includes indicators such as weight loss, diarrhea, and bloody stools, was used to evaluate the severity of chronic colitis induced in the mouse model, to monitor the animal health status, and to evaluate the effect of the interventions. The results showed significant weight changes across all groups after DSS induction (Figure 1A). Diarrhea was most severe in the IND group, and the curcuminoid groups significantly alleviated diarrhea, though not to the level of the CON group, with softer or unformed stools (Figure 1B). All DSS-treated groups exhibited persistent bloody stools or anal bleeding after the first DSS cycle, continuing until the experiment’s end (Figure 1C,D). However, the curcuminoid-treated groups effectively reduced bleeding, indicating that curcumin and BDMC improved symptoms of bloody stools and anal bleeding. Based on the DAI value (calculated as the average of four scores) analysis, which revealed significantly lower values in the sample groups compared to the DSS group (Figure 1E), the results from the animal model indicate that interventions with 0.5% curcumin and both 0.5% and 0.1% BDMC significantly improved the clinical symptoms of DSS-induced colitis in mice. During DSS-induced chronic colitis, the intestinal wall undergoes repeated inflammation and healing, leading to mucosal dysplasia, crypt structure disruption, lymphocyte infiltration, and fibrosis, ultimately causing colon shortening and thickening.^20^ In this study, DSS significantly shortened the colon, while the curcuminoid-treated groups had significantly longer colons than the IND group (Figure 2A,B), suggesting that the samples might alleviate mucosal dysplasia, thus preventing colon shortening. This finding was further supported by H&E-stained tissue sections (Figure 2C).

The severity of colitis can be assessed by direct observation of the intestine or by interpreting H&E-stained tissue sections under a microscope. Typical DSS-induced histological changes include goblet cell damage, epithelial ulcers, and neutrophil infiltration into the submucosa and lamina propria.^21^ In these sections, the DSS group showed significant submucosal and muscular layer expansion, neutrophil infiltration in the lamina propria, and a disordered epithelial goblet cell arrangement. Although other groups still had immune cell infiltration in the lamina propria, their epithelial structures were more intact, with 0.5% BDMC being the most effective, followed by 0.1% BDMC. The 0.5% CUR group, despite having a more intact epithelial structure, exhibited inflammatory thickening, suggesting that curcumin and BDMC may relieve colitis by maintaining intestinal barrier integrity, with BDMC being more effective than curcumin as it can effectively protect the intestinal epithelial structure and mitigate inflammation-induced tissue proliferation.

### 3.2 BDMC and Curcumin Protected Mucosal Integrity by Modulating TJ Proteins in the Colonic Tissue

**Figure 3:**
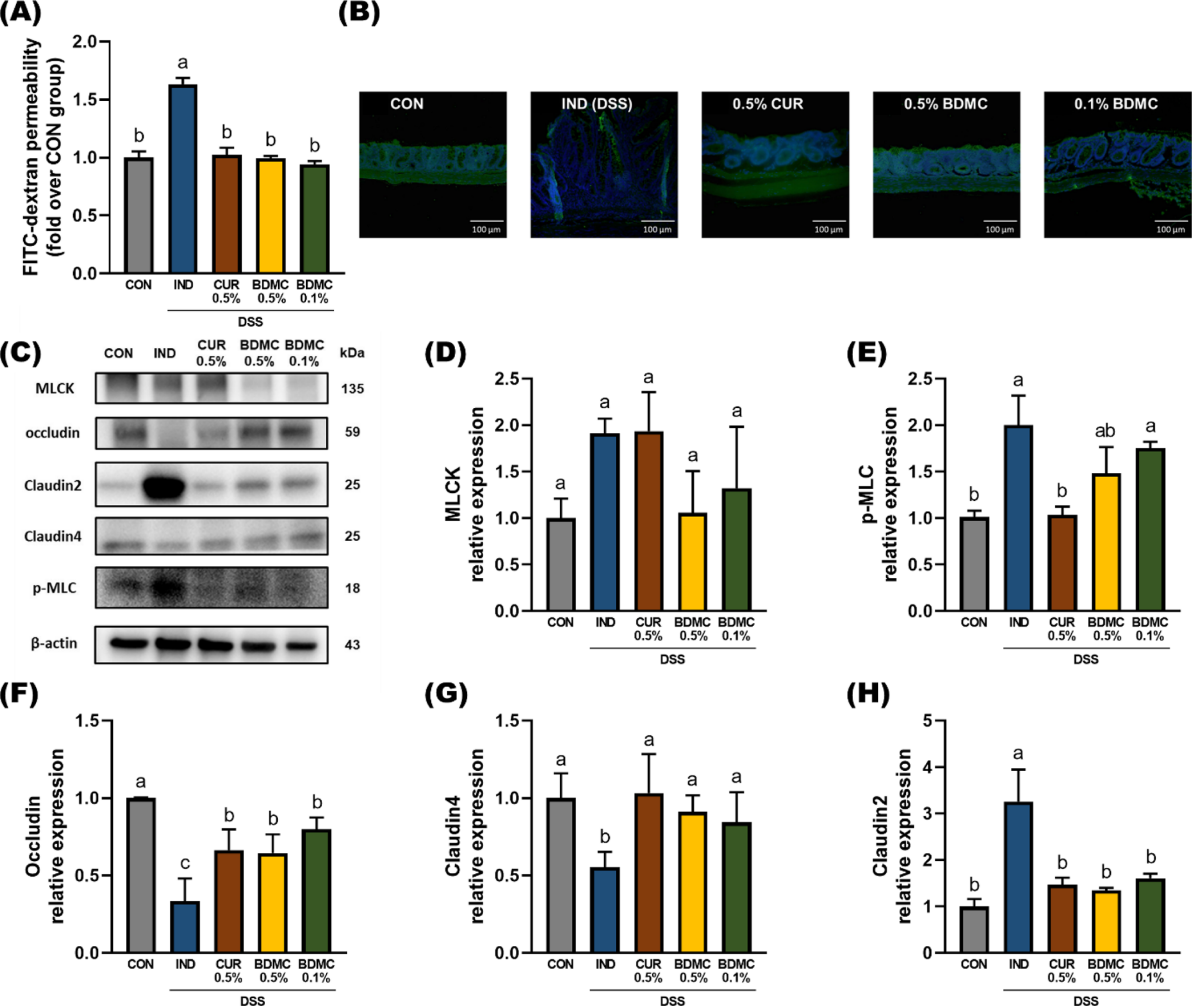
Effects of BDMC and curcumin on gut tight junction expression in DSS-induced ICR mice. (A) Gut permeability was assessed by measuring levels of FITC-dextran in the serum after oral gavage of 4 kDa FITC-dextran for 4 h. (B) The distribution of zonula occludens-1 (ZO-1) and DAPI (nuclei) in the colonic tissue sections was detected by immunofluorescence staining and representative photomicrographs at 100 × magnification (scale bar: 100 μm). (C) Representative protein expression of MLCK, p-MLC, occludin, claudin-4, and claudin-2. The relative expressions of (D) MLCK, (E) p-MLC, (F) occludin, (G) claudin-4, and (H) claudin-2 were normalized to β-actin and quantified by ImageJ. Data are presented as mean ± SEM, *n* = 3 per group, and *p* values were determined by (D–H) one-way ANOVA with subsequent Duncan’s multiple comparison test. The values with different letters are significantly different (*p* < 0.05) between each group.

Research suggests that increased intestinal barrier permeability may occur before the onset of clinical symptoms of IBD, making the maintenance of barrier integrity a potential strategy for early prevention.^22^ Data from the previous sections show that both curcumin and BDMC significantly alleviate DSS-induced clinical symptoms, reduce tissue damage, and prevent colon shortening. Therefore, this study further investigated whether curcumin and BDMC can protect the intestinal barrier from damage. In an FITC-dextran permeability test, fasting mice were administered FITC-dextran, and its concentration in the blood was measured 4 h later as a marker of intestinal permeability. The results showed that blood FITC-dextran levels were significantly reduced in the curcuminoid-treated groups, indicating that curcumin and BDMC effectively protect the intestinal barrier at these concentrations (Figure 3A).

Paracellular transport pathways are regulated by intercellular junctions, mainly composed of two protein complexes located at the apical membrane, known as the apical junctional complex. This complex includes tight junction proteins and adherens junctions. Tight junction proteins, such as ZO, occludin, and claudin, are located at the apical end of adjacent epithelial cells and govern paracellular permeability. They play a critical role in maintaining intestinal barrier function and facilitating immune response, digestion, and nutrient absorption while also defending against external microorganisms, antigens, and bacteria. Research indicates that abnormalities in tight junction function can lead to metabolic or inflammatory diseases.^23^ In immunofluorescence staining (Figure 3B), it was observed that DSS caused the degradation of the ZO-1 protein, marked by green fluorescence, in the intestinal epithelial cells of mice, leading to intestinal damage. However, the intervention with BDMC and curcumin was able to stabilize ZO-1 and maintain the integrity of the intestinal framework.

Studies have shown that TNF-α activates NF-κB, which translocates into the nucleus and binds to the promoter region of the myosin light-chain kinase (MLCK) gene, leading to increased expression of MLCK mRNA and protein. This, in turn, phosphorylates the myosin light chain (p-MLC), promoting cytoskeletal contraction and affecting the stability of intestinal tight junctions. In IBD, excessive activation of this pathway leads to the disruption of tight junctions in intestinal epithelial cells, increasing intestinal permeability and allowing inflammation to spread more easily.^24^ To assess the health of the intestinal barrier, this study tested MLCK, p-MLC, and a range of tight junction proteins, such as occludin, claudin-4, and claudin-2 (Figure 3C). MLCK protein expression was elevated in the IND and 0.5% CUR groups, but there was no significant difference between the CON and BDMC groups (Figure 3D). The IND group showed significantly higher p-MLC protein expression than the CON group, indicating inflammation-induced damage to the intestinal barrier. The 0.5% CUR group effectively reduced p-MLC expression, while 0.5% BDMC showed no significant difference compared to the CON and IND groups, and 0.1% BDMC showed no protective effect (Figure 3E).

Previous research indicates that 4 days of DSS treatment in mice reduces occludin expression, leading to intestinal barrier damage and worsening colitis.^25^ Clinically, ulcerative colitis patients in the acute phase also show lower claudin-4 expression compared to healthy individuals.^26^ The experimental results indicate that curcuminoid treatment effectively mitigates DSS-induced tight junction protein disruption. The expression of occludin in the IND group was significantly lower than in the CON and curcuminoid groups. Both concentrations of BDMC and 0.5% curcumin significantly increased the level of the CON group, but it did not reach the level of the CON group (Figure 3F). Claudin-4 expression was significantly reduced in the IND group due to DSS, but all curcuminoid-treated groups significantly increased the level of claudin-4 expression (Figure 3G). Claudin-2, involved in sodium and calcium ion transport, shows an inverse relationship with transepithelial electrical resistance (TEER), indicating that increased claudin-2 is closely related to barrier leakage. Data showed that the curcuminoid treatments effectively reduced the level of DSS-induced upregulation of claudin-2 expression (Figure 3H).

### 3.3 Inhibitory Effect of BDMC and Curcumin on Proinflammatory Responses in DSS-Induced Mice

**Figure 4:**
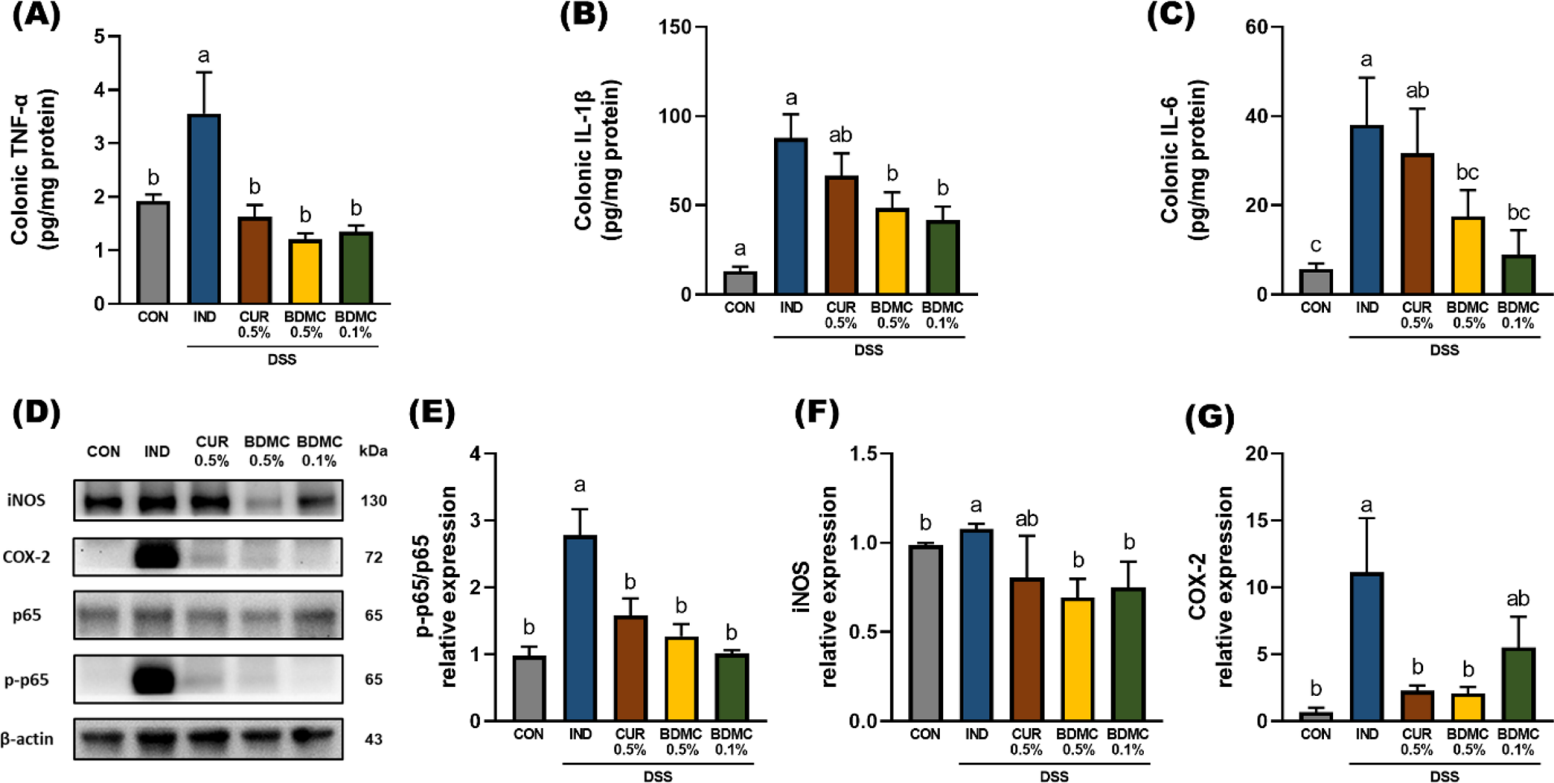
Effects of BDMC and curcumin on colonic cytokine production and attenuated NF-κB activation in DSS-induced ICR mice. (A–C) The production of TNF-α, IL-1β, and IL-6 in colonic tissue was analyzed by ELISA kit (*n* = 3–6 per group). (D) Representative protein expression of iNOS, p65, p-p65, and COX-2. The relative expressions of (E) p65 and p-p6, (F) iNOS, and (G) COX-2 were normalized to β-actin and quantified by ImageJ. Data are presented as mean ± SEM, *n* = 3 per group, and *p* values were determined by one-way ANOVA with subsequent Duncan’s multiple comparison test. The values with different letters are significantly different (*p* < 0.05) between each group.

The development of IBD is triggered by intestinal barrier damage and an excessive immune response. DSS-induced colitis first damages the epithelial barrier, which then enhances the accumulation of immune cells in the inflamed area, leading to excessive cytokine secretion and sustained intestinal inflammation. During inflammation, large amounts of cytokines like TNF-α, IL-1β, and IL-6 are released, activating NF-κB, which further affects the TJ structure and increases intestinal permeability, disrupting the normal function of the mucosal barrier.^27^ To determine whether curcuminoids could strengthen TJ expression by modulating inflammation, this study evaluated cytokine concentrations in intestinal tissues. The results showed that in the DSS-treated group, TNF-α, IL-1β, and IL-6 levels were significantly increased. In the curcuminoid-treated groups, BDMC at both 0.5% and 0.1% concentrations significantly reduced these cytokine levels, while 0.5% CUR had only a limited effect on IL-1β and IL-6. This suggests that the curcuminoids may have potential anti-inflammatory effects (Figure 4A, B,C).

According to the data from Section 3.2, curcumin and BDMC effectively support the TJ function, which may be related to their regulation of inflammatory pathways. Previous studies have indicated that TNF-α promotes NF-κB activation, leading to increased MLCK expression and MLC phosphorylation, which disrupt TJ structure.^28^ Additionally, TNF-α and IL-1β levels are increased due to positive regulation by NF-κB. To further investigate this mechanism, the study used Western blotting to detect the expression of p65, p-p65, iNOS, and COX-2. The data showed that all curcuminoid-treated groups significantly reduced DSS-induced upregulation of p-p65 expression, indicating that the curcuminoids effectively reduced p65 phosphorylation, further protecting TJs from damage (Figure 4E).

When macrophages are activated by bacterial invasion, they produce chemokines to attract leukocytes to the site of infection, triggering an inflammatory response. These cells release monocyte chemoattractant protein-1 (MCP-1) to recruit more monocytes, which then differentiate into macrophages or dendritic cells in the tissue. Macrophages increase the expression of iNOS and COX-2, promoting the infiltration of inflammatory substances and immune cells into the tissue and enhancing the oxidative inflammatory responses to combat bacteria. However, if this immune response is too strong or lasts too long, it can cause intestinal tissue damage, exacerbate inflammation, and may lead to excessive immune cell accumulation and worsening colitis.^29^

The results showed that in the DSS-induced IND group, iNOS and COX-2 protein expression levels were significantly increased, indicating a large infiltration of neutrophils into the tissue and localized inflammation. Both 0.5% and 0.1% BDMC interventions effectively reduced the level of DSS-induced upregulation of iNOS expression (Figure 4F), while both 0.5% CUR and BDMC interventions effectively reduced the level of DSS-induced upregulation of COX-2 expression (Figure 4G). This suggests that the curcuminoids effectively mitigate the increased expression of iNOS and COX-2, with 0.5% BDMC showing the best results, thereby alleviating DSS-induced intestinal inflammation.

### 3.4 BDMC and Curcumin Inhibited Epithelial Cell Apoptosis in Colonic Tissue

As described in Section 3.3, the function of the intestinal barrier in IBD patients is influenced not only by the immune response but also by TNF-α through the p-MLC pathway. This pathway can rapidly disrupt TJs, leading to increased permeability between epithelial cells.^30^ Moreover, abnormalities in apoptosis can increase the rate of cell shedding, outpacing their regeneration and thus compromising the stability of the barrier, making the intestine less effective at blocking harmful external substances. Previous studies have shown that mice lacking the MLCK gene can resist TJ abnormalities caused by cytokines but cannot prevent intestinal barrier damage due to apoptosis.^31^

Apoptosis is regulated by both intrinsic and extrinsic pathways, with caspase-3 and caspase-7 being key proteins involved in both processes. The activation of caspase-3 and caspase-7 is a crucial step in triggering apoptosis when TNF-α induces cell loss. Additionally, the Bax/Bcl-2 ratio is considered a critical indicator of apoptosis, with Bax being a pro-apoptotic and Bcl-2 an antiapoptotic protein.

**Figure 5:**
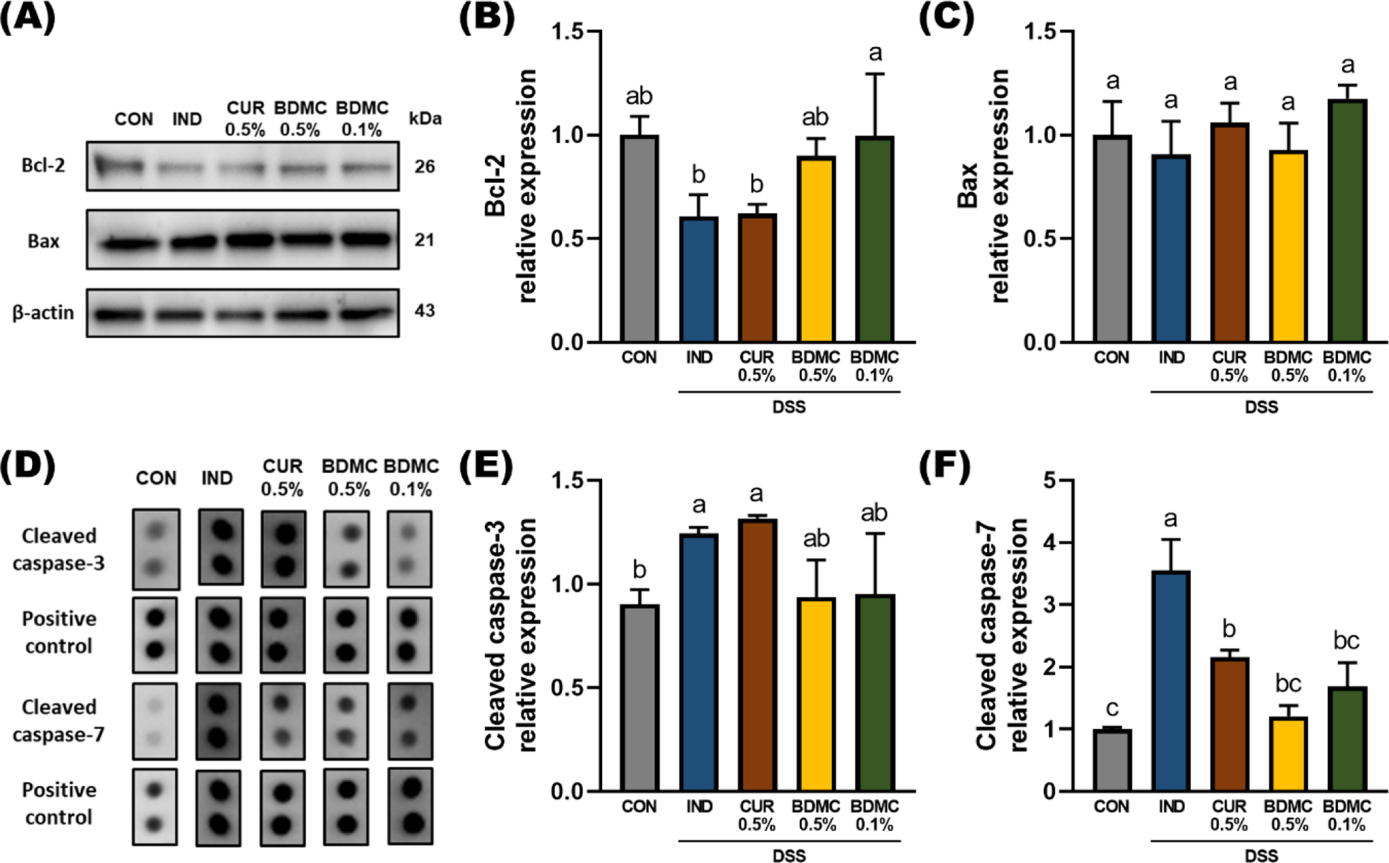
Effects of BDMC and curcumin on colonic epithelial cell apoptosis in DSS-induced ICR mice. (A) Representative protein expression of (B) Bcl-2 and (C) Bax protein, where relative expressions are normalized to β-actin and quantified by ImageJ. (D) Representative protein expression of (E) cleaved caspase-3 and (F) cleaved caspase-7 protein, where relative expressions were normalized to positive control and quantified by ImageJ. Data are presented as mean ± SEM, *n* = 3 per group, and *p* values were determined by one-way ANOVA with subsequent Duncan’s multiple comparison test. The values with different letters are significantly different (*p* < 0.05) between each group.

In this study, Western blotting and protein microarrays were used to examine the expression of apoptosis-related proteins in the mouse colon. The results showed no significant difference in Bax protein levels between the CON, IND, and sample groups (Figure 5B). However, 0.1% BDMC effectively increased the level of Bcl-2 protein expression. Although 0.5% CUR and BDMC did not show a significant increase, they did exhibit a trend toward higher expression (Figure 5C). Previous studies have extensively discussed the inhibitory effect of curcumin on Bcl-2 in cancer cells, and some studies have also reported that curcumin intervention can enhance Bcl-2 expression, thereby protecting organs from damage.^32,33^ Regarding caspase-3 and caspase-7, DSS was observed to increase the activation of both proteins (Figures 5E,F). While 0.5% CUR did not significantly reduce their activation, 0.5% and 0.1% BDMC effectively decreased the activation of caspase-7 (Figure 5F), suggesting that BDMC may help maintain the stability of tight junctions by reducing apoptosis in intestinal epithelial cells.

### 3.5 BDMC and Curcumin Regulated the Gut Microbiota Compositions and Short-Chain Fatty Acids in Colitic Feces

**Figure 6:**
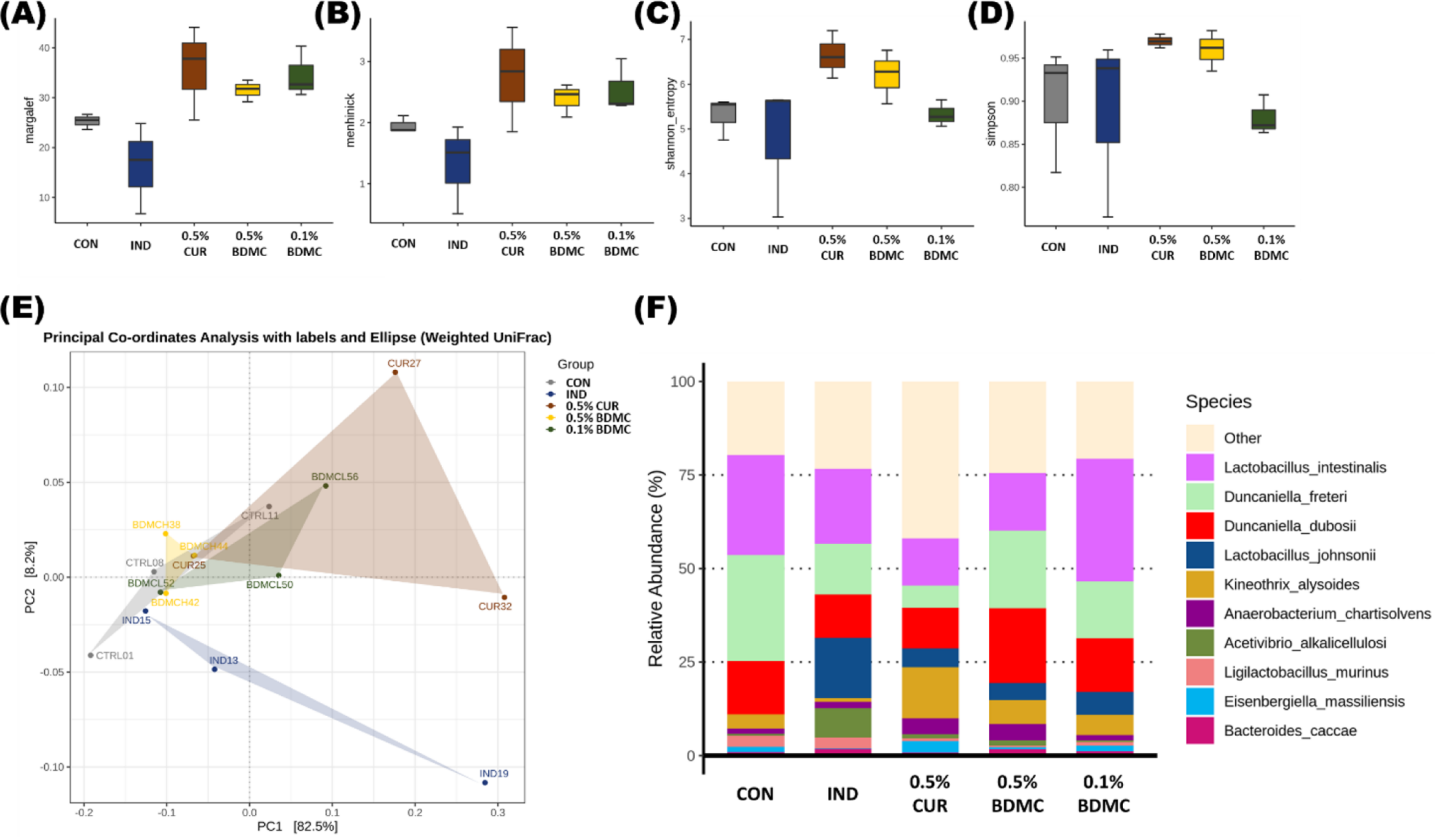
Effects of BDMC and curcumin on alpha and beta diversity indices and relative abundance at species level of gut microbiota in DSS-induced ICR mice. (A) Menhinick’s richness index, (B) Margalef’s richness index, (C) Shannon diversity index, and (D) Simpson diversity index. Each box plot represents the median, interquartile range, and minimum and maximum values, *n* = 3 per group. (E) Principle coordinate analysis (PCoA) plot based on weighted UniFrac distance metric. Each point represents one sample, and different colors represent the different groups, *n* = 3 per group. (F) Relative abundance of the top 10 bacteria at the species level of taxonomy.

Alpha diversity is used to measure biodiversity within a single ecosystem. The Menhinick and Margalef indices estimate species richness based on the total number of species and the total number of samples. The indices of the three curcuminoid intervention groups are relatively similar, showing higher species richness compared to the CON and IND groups (Figure 6A,B). Additionally, the Shannon and Simpson indices are used to assess biodiversity. According to the analysis of these indices, the microbial diversity among the five groups showed no significant differences (Figure 6C,D). Beta diversity is used to evaluate differences in microbial composition between different groups. The results indicated that the 0.5% BDMC group showed the closest similarity to the CON group, with the 0.1% BDMC group following closely. In contrast, although the 0.5% CUR group had some overlap with the CON group, it demonstrated greater variation within the group, highlighting the consistency of BDMC’s effects. The IND group, however, showed more divergence from the CON group, indicating more distinct differences in the microbial composition (Figure 6E). Previous studies have reported that, compared to acute colitis, the decline in alpha diversity in DSS-induced IBD is less pronounced.^34^ This may be due to the rapid recovery of the gut microbial composition to a healthier state during inflammation remission. Additionally, past research on DSS-induced colitis in animal models with curcumin intervention showed a similar Shannon index to the results of this study.^35^

Studies involving probiotic intervention in DSS-induced colitis in mice have observed that *Lactobacillus intestinalis* can serve as a marker species for probiotic consumption. Most studies also suggest that it effectively inhibits the growth of pathogenic bacteria.^36^ The relative abundance bar chart (Figure 6F) shows that *L. intestinalis* is higher in the CON and 0.1% BDMC groups and decreases in the IND, CUR, and 0.5% BDMC groups. This indicates that DSS induction and higher concentrations of the curcuminoids may inhibit the growth of this species, suggesting it may not be the primary factor in the improvement of colitis by curcumin and BDMC. There is limited literature on *Duncaniella freteri* and *Duncaniella dubosii*, but some studies have reported that the abundance of the *Duncaniella* genus is negatively correlated with the severity of colitis.^37^ In this study, the relative abundance of these species was lower in the IND group, and the CUR may reduce the abundance further. Conversely, both 0.5% BDMC and 0.1% BDMC groups were able to increase their abundance. *Lactobacillus johnsonii* has been identified as one of the key species that helps protect the gut from fungal invasion during periods of mucosal damage.^38^ In this study, *L. johnsonii* was present at very low levels in the CON group, while its abundance increased in the DSS-induced groups, particularly in the IND group, perhaps due to the influence of gut repair processes. *Kineothrix alysoides* is a butyrate-producing species and is considered a gut probiotic.^39^ In this study, the IND group showed a decrease in its relative abundance, while the curcuminoid groups effectively increased its abundance. Overall, the relative abundance bar chart indicates that curcumin and BDMC can effectively improve DSS-induced gut microbiota dysbiosis, with the gut microbiota composition in the BDMC-treated groups being more similar to that of the CON group. Based on the gut microbiota results, both curcumin and BDMC interventions increased the relative abundance of *Kineothrix alysoides*.

**Figure 7:**
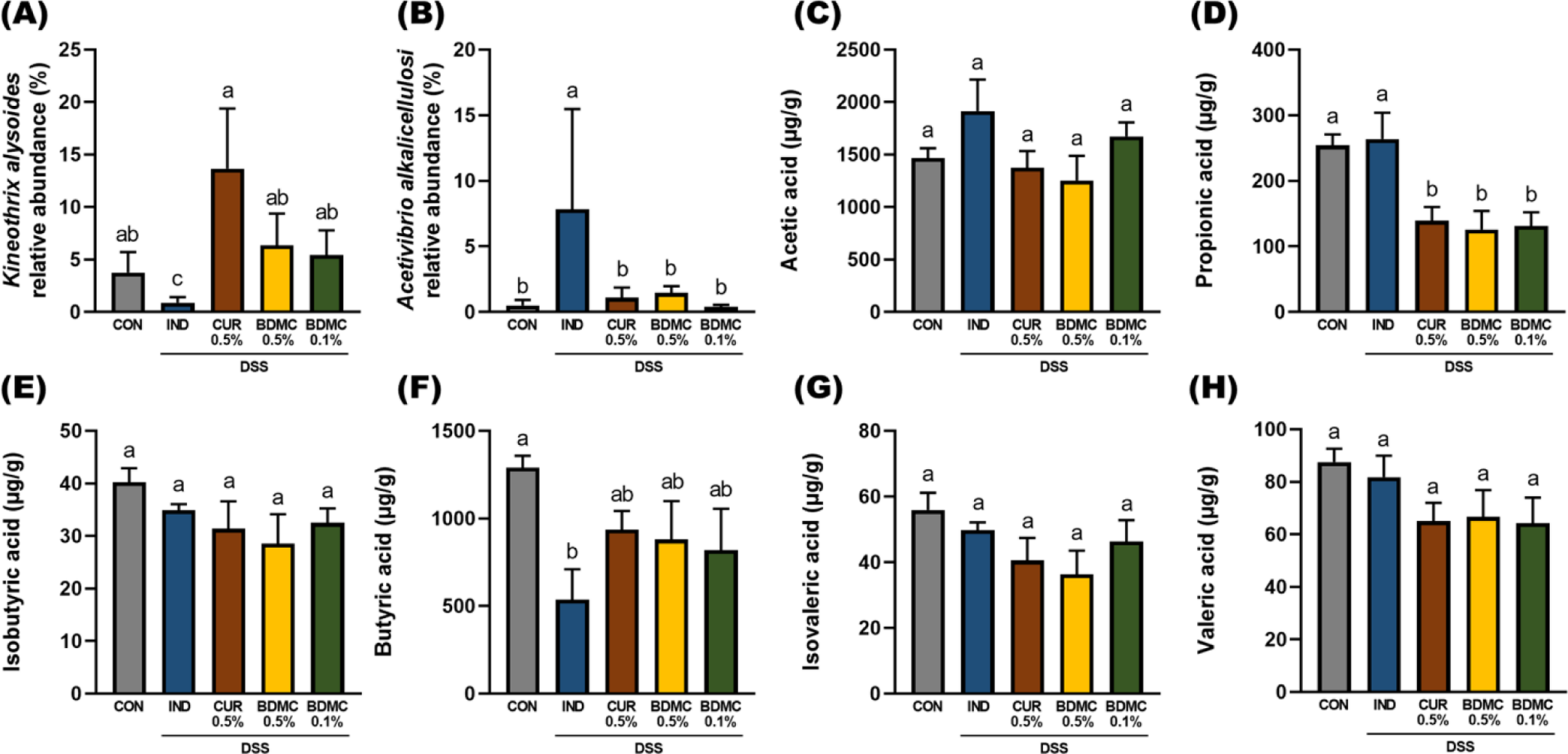
Effects of BDMC and curcumin on microbiota relative abundance and feces short-chain fatty acid content. Feces were collected from mouse colons after sacrifice. Relative abundance of (A) *Kineothrix alysoides* and (B) *Acetivibrio alkalicellulosi*. The contents of (C) acetic acid, (D) propionic acid, (E) isobutyric acid, (F) butyric acid, (G) isovaleric acid, and (H) valeric acid were analyzed by GC–MS. Data are presented as mean values ± SEM, *n* = 6 per group, and *p* values were determined by one-way ANOVA with subsequent Duncan’s multiple comparison test. The values with different letters are significantly different (*p* < 0.05) between each group.

In addition, we measured the distribution of short-chain fatty acids (SCFAs) in the intestinal feces to determine whether the curcuminoids promoted SCFA biosynthesis in mice by modulating the gut microbiota (Figure 7). This would provide an energy source for the intestinal mucosa and help alleviate colitis. Regarding acetic acid, although there was no significant difference in its content, the IND group had higher levels of acetic acid in their feces, possibly due to the invasion of *Acetivibrio alkalicellulosi*. Regarding propionic acid content in the feces, the curcuminoid groups showed a significant decrease. Butyric acid is considered one of the most important SCFAs due to its roles in energy supply, TJ strengthening, anti-inflammatory effects, and inhibition of tumor growth.^40^ The gut microbiota results showed that CUR and BDMC interventions increased the relative abundance of *Kineothrix alysoides*. In this study, the curcuminoid interventions increased the butyric acid content, although not significantly, which is consistent with the microbiota results. The contents of isobutyric acid, isovaleric acid, and valeric acid were low in this study, with consistent trends showing decreased SCFA content after curcuminoid intervention but without significant differences. Overall, after curcuminoid intervention, there was a decrease in some SCFAs besides butyric acid, which contrasts with findings in the literature.^41^

Some studies indicate that approximately 90–95% of SCFAs are absorbed and metabolized by the intestinal mucosa, with only 5–10% excreted through feces.^42^ The impaired intestines of IBD patients may reduce SCFA absorption, leading to more SCFAs being excreted in feces. Other studies suggest that fecal SCFA levels are positively correlated with the severity of DSS induction.^43^ Additionally, fecal SCFA levels in mice with diarrhea-predominant irritable bowel syndrome (IBS) are typically higher than in constipation-predominant mice, indicating that fecal SCFA levels are not only associated with gut microbiota but may also be influenced by colonic transit time.^44^ In this experiment, the mice in the IND group were still recovering from diarrhea at the time of sacrifice, which may explain the lack of significant differences in fecal SCFAs compared to the CON group. Additionally, the intestinal barrier in the curcuminoid-treated group was more intact, which may have enhanced the absorption of SCFAs in the colon, resulting in no significant increase in SCFAs in the feces.

### 3.6 Distribution of BDMC and Curcumin in Feces and Tissues

As previously mentioned, although in this study curcumin and BDMC have shown anti-inflammatory and TJ strengthening effects, past research indicates that even when humans consume 8 g of curcumin daily, the plasma concentration of curcumin remains only around 2.5 ng/mL.^45^ This is primarily due to curcumin’s low water solubility and bioavailability as it may be broken down or converted into metabolites by the digestive system.^46^ Therefore, this study further explored the levels of curcumin and BDMC in the feces and organs.

After extracting the feces and organs, analysis was conducted using LC–MS/MS. The results showed that a large amount of curcumin (743.07–1675.78 μg/g) was excreted through feces, which aligns with the literature.^47^ BDMC, on the other hand, was absorbed more efficiently, resulting in significantly less excretion in feces (Table 1). BDMC also showed lower levels in the liver and kidneys, likely due to its shorter retention time in the body, which is consistent with findings from previous studies on the bioavailability of curcumin and BDMC in rats.^48^

**Table 1:** Content of Curcumin, Curcumin Metabolites, and BDMC in Mouse Feces and Organs^a^

		Component amount **(μg/g)**				
Sample	Group	CUR	BDMC	THC	HHC	FER
Feces	0.5% CUR	1245.81 ± 330.42	N.D.	9.63 ± 4.09	0.38 ± 0.19	12.58 ± 6.14
	0.5% BDMC	N.D.	223.24 ± 119.59	N.D.	N.D.	N.D.
	0.1% BDMC	N.D.	78.92 ± 37.92	N.D.	N.D.	N.D.
Liver	0.5% CUR	0.55 ± 0.47	N.D.	0.15 ± 0.08	1.75 ± 0.62	N.D.
	0.5% BDMC	N.D.	0.15 ± 0.17	N.D.	N.D.	N.D.
	0.1% BDMC	N.D.	N.D.	N.D.	N.D.	N.D.
Kidney	0.5% CUR	0.31 ± 0.25	N.D.	0.05 ± 0.11	0.24 ± 0.21	N.D.
	0.5% BDMC	N.D.	0.09 ± 0.12	N.D.	N.D.	N.D.
	0.1% BDMC	N.D.	N.D.	N.D.	N.D.	N.D.

a N.D. = not detected, below limit of detection. Data are presented as mean values ± SEM, *n* = 4 per group.

Additionally, this study analyzed the *in vivo* distribution of some curcumin metabolites and degradation products. Interestingly, considerable amounts of tetrahydrocurcumin (THC) and hexahydrocurcumin (HHC) were found in feces, liver, and kidneys. Previous studies have noted that curcuminoid metabolites primarily exist in the form of sulfates and glucuronides but may also be metabolized into THC and HHC. Due to their structural properties, THC and HHC have significantly enhanced water solubility and exhibit strong antioxidant effects, which can help alleviate oxidative kidney damage.^49^ Feruloylacetone (FER), a degradation product and microbial metabolite of curcumin, has also been shown to have anti-inflammatory and anti-TNF-α effects.^50^ In the CUR group, although most curcumin was excreted through feces, a variety of potentially beneficial compounds were generated through metabolism and degradation, suggesting that some degree of curcumin’s efficacy may be derived from these metabolites and degradation products.

### 3.7 BDMC and Curcumin as Potential Strategies for Mitigating Inflammatory Bowel Disease

This study investigated the effects of BDMC and curcumin on DSS-induced colitis in mice, focusing on their roles in reducing inflammation and enhancing intestinal barrier function. Both 0.5% curcumin and BDMC, along with 0.1% BDMC, significantly improved clinical symptoms, such as weight loss, stool consistency, and intestinal bleeding. Histological analyses confirmed their protective effects on intestinal epithelial structures by enhancing tight junction proteins such as occludin and claudin-4 while reducing claudin-2 levels, thereby decreasing permeability.

**Figure 8:**
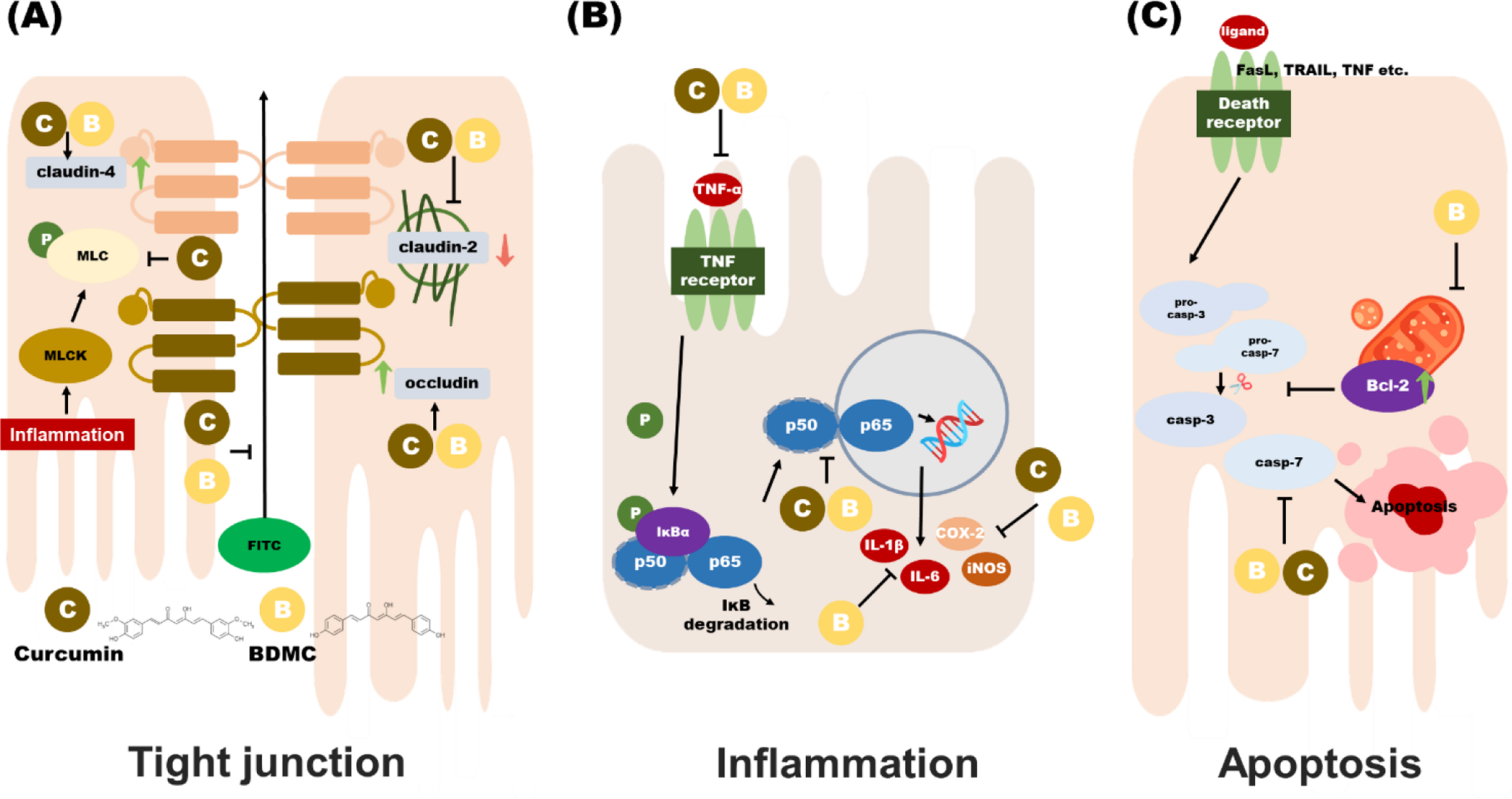
Molecular mechanisms of BDMC and curcumin on ameliorating colitis in DSS-induced ICR mice. The molecular mechanisms by which BDMC and curcumin alleviate DSS-induced colitis in ICR mice are categorized into three pathways: (A) tight junction-related pathways, (B) inflammation-related pathways, and (C) apoptosis-related pathways.

BDMC and curcumin effectively suppressed inflammatory pathways, reducing cytokines like TNF-α, IL-1β, and IL-6 and lowering NF-κB phosphorylation. BDMC showed superior antiapoptotic effects by reducing cleaved caspase-3 and enhancing Bcl-2 expression, providing greater protection against cell apoptosis (Figure 8). Gut microbiota analysis revealed that BDMC restored microbial balance and increased butyrate-producing bacteria such as *Kineothrix alysoides*, particularly at 0.5% concentration, with higher bioavailability compared to curcumin.

Currently, the guidelines of the Taiwan Herbal Pharmacopoeia and The Pharmacopoeia of the People’s Republic of China 2020 Edition, two official compendiums of Chinese drugs, state that medicinal turmeric must contain more than 1% curcumin.^17^ Curcumin is considered the primary active component in turmeric. However, this study suggests that secondary components like BDMC, despite being present in lower concentrations, may offer comparable or even superior efficacy. Compared to curcumin, BDMC shows greater potential in protecting intestinal barrier integrity and combating inflammation, making it a key phytochemical for the future prevention or treatment of IBD.

## Abbreviations

Bcl-2: B-cell lymphoma 2
Bax: Bcl-2-associated X protein
BDMC: Bisdemethoxycurcumin
COX-2: Cyclooxygenase-2
CUR: Curcumin
DMC: Demethoxycurcumin
DSS: Dextran sulfate sodium
DAI: Disease activity index
ELISA: Enzyme-linked immunosorbent assay
FER: Feruloylacetone
FITC: Fluorescein isothiocyanate
GC–MS: Gas chromatography–mass spectrometry
H&E: Hematoxylin and Eosin
HHC: Hexahydrocurcumin
iNOS: Inducible nitric oxide synthase
IBD: Inflammatory bowel disease
IL: Interleukin
IBS: Irritable bowel syndrome
LC–MS/MS: Liquid chromatography–tandem mass spectrometry
MAPK: Mitogen-activated protein kinase
MLCK: Myosin light chain kinase
NF-κB: Nuclear factor kappa-light-chain-enhancer of activated B cells
PI3K: Phosphoinositide 3-kinase
p-MLC: Phospho-myosin light chain 2
Akt: Protein kinase B (PKB)
SCFA: Short-chain fatty acid
THC: Tetrahydrocurcumin
TJ: Tight junction
TNF-α: Tumor necrosis factor alpha
ZO-1: Zonula occludens-1
